# Video‐assisted thoracoscopic surgery lobectomy might be a feasible alternative for surgically resectable pathological N2 non‐small cell lung cancer patients

**DOI:** 10.1111/1759-7714.13680

**Published:** 2020-11-18

**Authors:** Jinbo Zhao, Weimiao Li, Meng Wang, Lunxu Liu, Xiangning Fu, Yin Li, Lin Xu, Yang Liu, Heng Zhao, Jian Hu, Deruo Liu, Jianfei Shen, Haiying Yang, Xiaofei Li

**Affiliations:** ^1^ Department of Thoracic Surgery Tangdu Hospital, Fourth Military Medical University Xi'an China; ^2^ Department of Oncology The Second Affiliated Hospital of Xi'an Jiaotong University Xi'an China; ^3^ Department of Thoracic Surgery Tianjin Chest Hospital Tianjin China; ^4^ Department of Thoracic Surgery West China Hospital, Sichuan University Chengdu China; ^5^ Department of Thoracic Surgery Tongji Hospital, Tongji Medical College, Huazhong University of Science and Technology Wuhan China; ^6^ Department of Thoracic Surgery Henan Cancer Hospital Zhengzhou China; ^7^ Department of Thoracic Surgery Nanjing Medical University Affiliated Cancer Hospital, Cancer Institute of Jiangsu Province Nanjing China; ^8^ Jiangsu Key Laboratory of Molecular and Translational Cancer Research Cancer Institute of Jiangsu Province Nanjing China; ^9^ Department of Thoracic Surgery Chinese People's Liberation Army General Hospital Beijing China; ^10^ Department of Thoracic Surgery Shanghai Chest Hospital Shanghai China; ^11^ Department of Thoracic Surgery First Hospital Affiliated to Medical College of Zhejiang University Hangzhou China; ^12^ Department of Thoracic Surgery China‐Japan Friendship Hospital Beijing China; ^13^ Department of Thoracic Surgery Taizhou Hospital of Zhejiang Province, Wenzhou Medical University Linhai China; ^14^ Medical Affairs Linkdoc Technology Co, Ltd Beijing China

**Keywords:** Lobectomy, overall survival, pathological N2 non‐small cell lung cancer (pN2 NSCLC), perioperative outcomes, video‐assisted thoracic surgery (VATS)

## Abstract

**Background:**

The majority of previous studies of the clinical outcome of video‐assisted thoracoscopic surgery (VATS) versus open lobectomy for pathological N2 non‐small cell lung cancer (pN2 NSCLC) have been single‐center experiences with small patient numbers. The aim of this study was therefore to investigate these procedures but in a large cohort of Chinese patients with pathological N2 NSCLC in real‐world conditions.

**Methods:**

Patients who underwent lobectomy for pN2 NSCLC by either VATS or thoracotomy were retrospectively reviewed from 10 tertiary hospitals between January 2014 and September 2017. Perioperative outcomes and overall survival of the patients were analyzed. Cox regression analysis was performed to identify potential prognostic factors. Propensity‐score analysis was performed to reduce cofounding biases and compare the clinical outcomes between both groups.

**Results:**

Among 2144 pN2 NSCLC, 1244 patients were managed by VATS and 900 by open procedure. A total of 305 (24.5%) and 344 patients died during VATS and the thoracotomy group during a median follow‐up of 16.7 and 15.6 months, respectively. VATS lobectomy patients had better overall survival when compared with those undergoing the open procedure (*P* < 0.0001). Multivariate COX regression analysis showed VATS lobectomy independently favored overall survival (HR = 0.75, 95% CI: 0.621–0.896, *P* = 0.0017). Better perioperative outcomes, including less blood loss, shorter drainage time and hospital stay, were also observed in patients undergoing VATS lobectomy (*P* < 0.05). After propensity‐score matching, 169 patients in each group were analyzed, and no survival difference were found between the two groups. Less blood loss was observed in the VATS group, but there was a longer operation time.

**Conclusions:**

VATS lobectomy might be a feasible alternative to conventional open surgery for resectable pN2 NSCLC.

**Key points:**

- Significant findings of the study: VATS lobectomy has comparative OS in pN2 NSCLC versus open procedure in resectable patients.
- What this study adds: VATS lobectomy might be feasible for pN2 NSCLC.

## Graphical abstract

The present study aimed to investigate the clinical outcome of video‐assisted thoracoscopic surgery (VATS) versus open lobectomy for pathological N2 non‐small cell lung cancer (pN2 NSCLC) using a large cohort of Chinese patients in real world conditions. VATS lobectomy might be a feasible alternative for pN2 NSCLC.

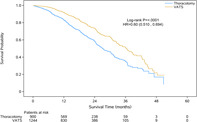

## Introduction

N2 positive non–small cell lung cancer (NSCLC) is a highly heterogeneous entity with regard to clinical outcome and patient management. Although the potential role of surgical resection in pathologically documented N2 (pN2) patients remains controversial,^1^ surgery is still considered as a major part of clinical practice and guideline recommendations for management of occult N2 or N2 disease after induction therapy. It has been reported to be associated with significant better overall survival in stage IIIA (N2) NSCLC patients when compared with nonsurgical interventions (three‐year overall survival: 42.1 ± 3.8% vs. 15.4 ± 1.5%, *P* < 0.0001).^2^ Moreover, the post resection five‐year overall survival has improved considerably during the past decades, from 19.9% in 1994 to 30.1%–44.7% in recent years,^3^, ^4^ highlighting the steadily improved outcomes of surgically managed pN2 NSCLC patients.

Video‐assisted thoracoscopic surgery (VATS), as a minimally invasive surgical technique, has emerged as a preferred alternative to conventional thoracotomy for early stage lung cancer.^5^ It has been demonstrated to be associated with significantly less blood loss and postoperative complications, as well as shorter hospital stay without compromising the overall survival of the patients.^6^, ^7^, ^8^ However, the risk of incomplete lymph node dissection and the missed opportunity of adjuvant chemotherapy due to unsuspected nodal metastasis during VATS has raised a major concern for its clinical use in pN2 patients.^9^ The current National Comprehensive Cancer Network (NCCN) guidelines recommend stopping surgery and administering induction therapy for patients with occult N2 nodes discovered when a VATS procedure is performed, although continuing the procedure is also suggested as an option. Nevertheless, the controversy remains as to whether VATS should be performed in NSCLC patients with N2 lymph node involvement. Some studies have indicated similar effects of VATS and open lobectomy on lymph node dissection^10^, ^11^ and clinical outcomes in occult N2 NSCLC patients (cN0‐pN2).^12^, ^13^, ^14^, ^15^, ^16^ In addition, VATS following neoadjuvant therapy has also been noted to be a safe and feasible procedure for stage IIIA and/or IIIB N2 NSCLC cases, whereas the opposite results of less lymph node dissection and increased risk of local recurrence have also been reported.^17^, ^18^ Most of these studies are single‐center experiences with small patient numbers. Thus, it remains unclear whether VATS can achieve similar outcomes in pN2 NSCLC patients when compared with the open procedure.

To bridge this knowledge gap, the aim of the present study was to investigate the clinical outcome of VATS and open approaches for pN2 NSCLC patients managed by lobectomy by board‐certified thoracic surgeons at the Departments of Thoracic Surgery of 10 tertiary hospitals in China between January 2014 and September 2017. A propensity‐score matching of clinical characteristics was also performed in the VATS and thoracotomy treated patients to reduce cofounding biases between the two groups. The study may provide insightful information about the performance of VATS versus thoracotomy in routine oncology clinical practice.

## Methods

### Study cohorts

**Figure 1:**
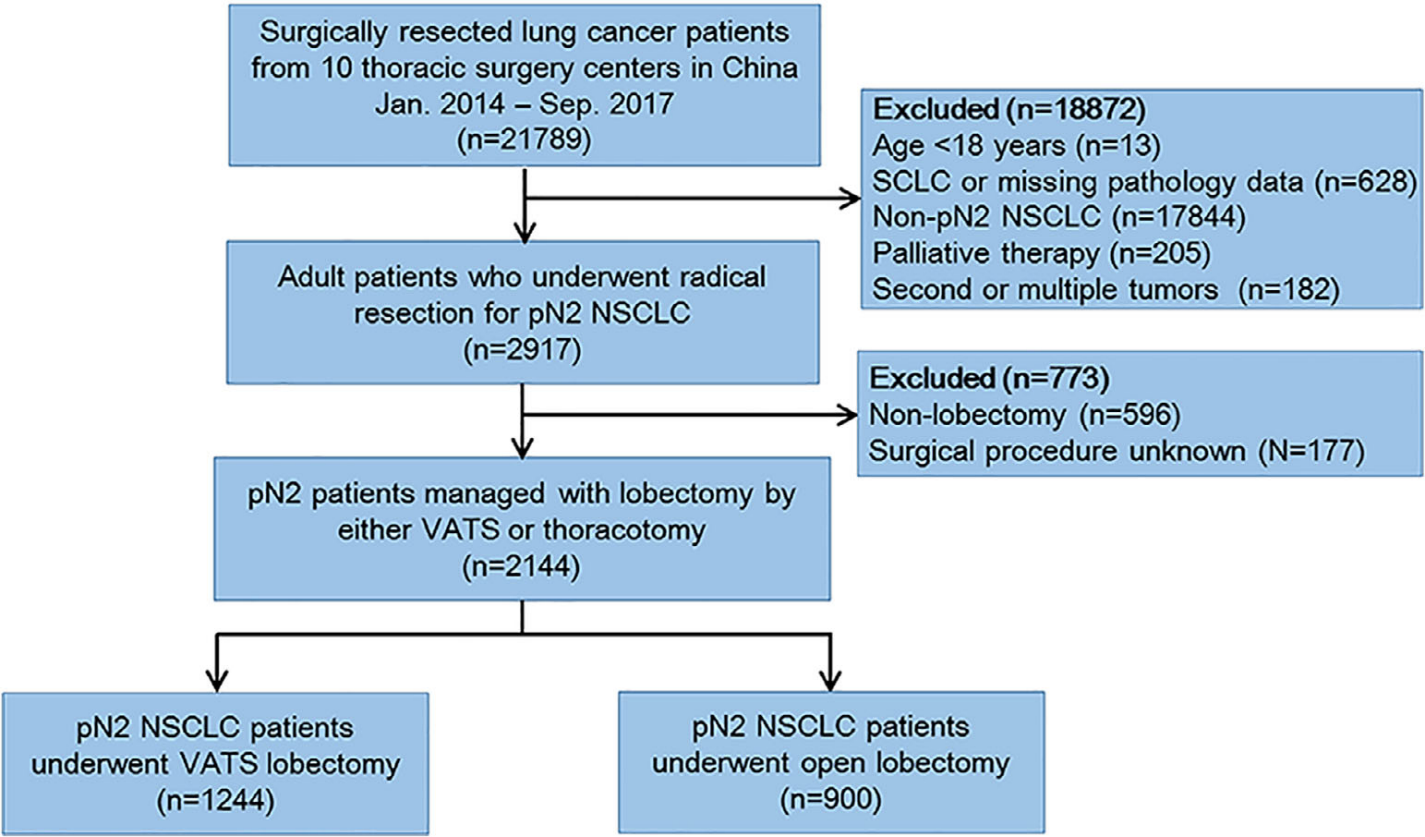
Flow chart of patient selection.

This was a multicenter retrospective cohort study. Lung cancer patients who had undergone surgical resection between January 2014 and September 2017 were retrospectively reviewed from the Department of Thoracic Surgery of 10 tertiary hospitals, which serve patients that cover almost 2/3 provinces and regions in China. Adult patients (≥18 years) who underwent complete resection and had postoperatively pathological N2 NSCLC were identified. Our study cohort was then further restricted to those who underwent lobectomy by either VATS or thoracotomy. Pneumonectomy and sleeve lobectomy as the other two important procedures for N2 NSCLC were not analyzed in this study due to the complexity of VATS. Patients converted to thoracotomy were excluded. We also excluded those with SCLC or missing data for histological type, as well as those with secondary primary tumors or multiple cancers. Patients were preoperatively evaluated according to routine oncology practice protocol, including contrast chest CT scan, abdominal CT or ultrasonography, bone single photon emission computed tomography, contrast brain MRI or CT scan, fiberoptic bronchoscopy, and pulmonary function tests. Positron emission tomography CT was only performed on highly suspicious patients.^19^, ^20^ The patient selection is described in Figure 1. The study protocol was approved by the Ethics Committee of all participating centers and was registered at ClinicalTrials.gov (NCT03613467). Written informed consent was waived for this retrospective study.

### Patient characteristics

A hybrid solution was introduced for data extracting from medical records based on a double reading/entry system.^21^ Clinical data on demographic and clinicopathological characteristics, and treatment were extracted from the LinkDoc database. Patient demographic characteristics included age at surgery, sex (male or female), smoking status (never, ever, or current), and neoadjuvant therapy delivered. Clinicopathological characteristics assessed were tumor laterality (right or left), histology (adenocarcinoma, squamous‐cell carcinoma, or other), pathological T (pT) stage (pT1, pT2, pT3 or pT4), as well as surgical margin status (R0 or non‐R0) and N2 lymph node station involved (single or multiple stations). Numbers of harvested and positive lymph nodes were also included and the ratio of positive lymph node was calculated.

### Clinical outcomes

Primary endpoint was overall survival of the patients calculated from the date of surgery to the date of death by any cause or last contact. Secondary endpoints were perioperative outcomes and risk factors for overall survival of the patients. Perioperative outcomes analyzed included operation time, estimated intraoperative blood loss and transfusion, duration of tube drainage and hospital stay, as well as postoperative mortality within 30 and 90 days. Follow‐up information was extracted from medical records or by telephone interview.

### Statistical analysis

Patient baseline characteristics and perioperative outcomes were summarized with descriptive statistics. Data were expressed as mean and standard deviation (SD), medians and ranges, or frequency and percentage when appropriate. Categorical variables were compared between the groups using a Chi‐square test, while continuous variables were analyzed using the Wilcoxon Rank sum test. Overall survival was estimated by Kaplan‐Meier method and compared between the groups using the log‐rank test. Sensitivity analysis was performed in patients who underwent neoadjuvant therapies or not and those with different pT stages to validate the results obtained in the overall populations. A Cox proportional hazards model was then established to determine potential risk factors for overall survival of patients.

Propensity scores were calculated using logistic regression method to adjust for potential confounders and indication biases between VATS and thoracotomy.^22^ The variables including sex (male or female), smoking history (yes or no), neoadjuvant therapy (yes or no), pT stage (pT1, pT2, pT3 or pT4), tumor location (right or left) and histology (adenocarcinoma, squamous‐cell carcinoma, or other) were chosen for propensity matching after removal of the potential factors that were not statistically significant in logistic regression analysis. The patients were then matched 1:1 between two groups using the nearest‐neighbor method.

All statistical analyses were two‐sided and a *P*‐value of less than 0.05 was considered statistically significant (SAS Version 9.4, SAS Institute, Cary, NC).

## Results

A total of 2144 patients who underwent lobectomy for pN2 NSCLC were identified, including 1244 patients managed by VATS and 900 by open procedure. The demographic and clinicopathological characteristics of the patients are summarized in Table 1. Several variables were imbalanced between both groups favoring the better survival of VATS patients, including a greater proportion of female and never smoking patients, lower rate of neoadjuvant chemotherapy delivered, greater percentage of earlier stage tumors, and a higher prevalence of patients with adenocarcinoma.

**Table 1:** Baseline characteristics of resected pN2 NSCLC patients

Variable	Thoracotomy (*n* = 900)	VATS (*n* = 1244)	*P*‐value
Median age, years (range)	60.1 (23.1–80.2)	59.6 (24.1–82.6)	0.6962
Sex, *n* (%)			<0.0001
Male	623 (69.2)	675 (54.3)	
Female	277 (30.8)	569 (45.7)	
Smoking status, *n* (%)^†^			<0.0001
Never	431 (48.8)	705 (59.0)	
Ever	222 (25.1)	232 (19.4)	
Current	230 (26.0)	257 (21.5)	
Tumor location, right *n* (%)	534 (59.3)	776 (62.4)	0.1533
pT staging, *n* (%)^†^			<0.0001
T1	114 (13.8)	385 (34.1)	
T2	472 (57.1)	644 (57.1)	
T3	150 (18.2)	77 (6.8)	
T4	90 (10.9)	22 (2.0)	
Pathology, *n* (%)			<0.0001
Adenocarcinoma	528 (58.7)	1015 (81.6)	
Squamous cell carcinoma	292 (32.4)	157 (12.6)	
Others	80 (8.9)	72 (5.8)	
Perioperative therapies, *n* (%)			0.0001
Neoadjuvant therapy	74 (8.2%)	46 (3.7%)	
Adjuvant therapy	546 (60.7%)	783 (62.9%)	
None	22 (2.4%)	37 (3.0%)	
Unknown	258 (28.7%)	378 (30.4%)	

^†^ Data not available for all patients.

pT, pathological T stage; VATS, video‐assisted thoracoscopic surgery.

Patients undergoing VATS lobectomy showed significantly better perioperative outcomes, including less intraoperative blood loss and lower rate of transfusion, shorter drainage time and hospital stay, as well as lower incidence of postoperative complications, except for longer surgical time (Table 2). The perioperative mortality rates for VATS lobectomy were 0.2% and 0.7% at postoperative days 30 and 90, when compared with 1.3% and 2.9% in thoracotomy group (*P* = 0.0028 and *P* < 0.0001), respectively.

**Table 2:** Perioperative outcomes of resected pN2 NSCLC patients

Variable	Thoracotomy (*n* = 900)	VATS (*n* = 1244)	*P*‐value
Operation time, minute	166.8 ± 63.5	180.5 ± 66.6	<0.0001
Estimated blood loss, mL	200 (0–6300)	100 (0–3500)	<0.0001
Intraoperative transfusion, *n* (%)	83 (9.2)	55 (4.4)	<0.0001
N2 station involvement, *n* (%)			<0.0002
Single station	422 (46.9)	685 (55.1)	
Multiple station	478 (53.1)	559 (44.9)	
Surgical margin, *n* (%)^†^			0.0810
R0	837 (93.9)	1180 (95.6)	
R1/R2	54 (6.1)	54 (4.4)	
Postoperative drainage, days	7.0 ± 5.2	6.5 ± 4.6	0.0079
Hospital stay, days	11.2 ± 6.3	9.7 ± 5.2	<0.0001
Perioperative complications, *n* (%)	82 (9.1)	82 (6.6)	0.0303
Perioperative mortality, *n* (%)			
30‐day	12 (1.3)	3 (0.2)	0.0028
90‐day	26 (2.9)	9 (0.7)	<0.0001

^†^ Data are not available for all patients.

VATS, video‐assisted thoracoscopic surgery.

**Figure 2:**
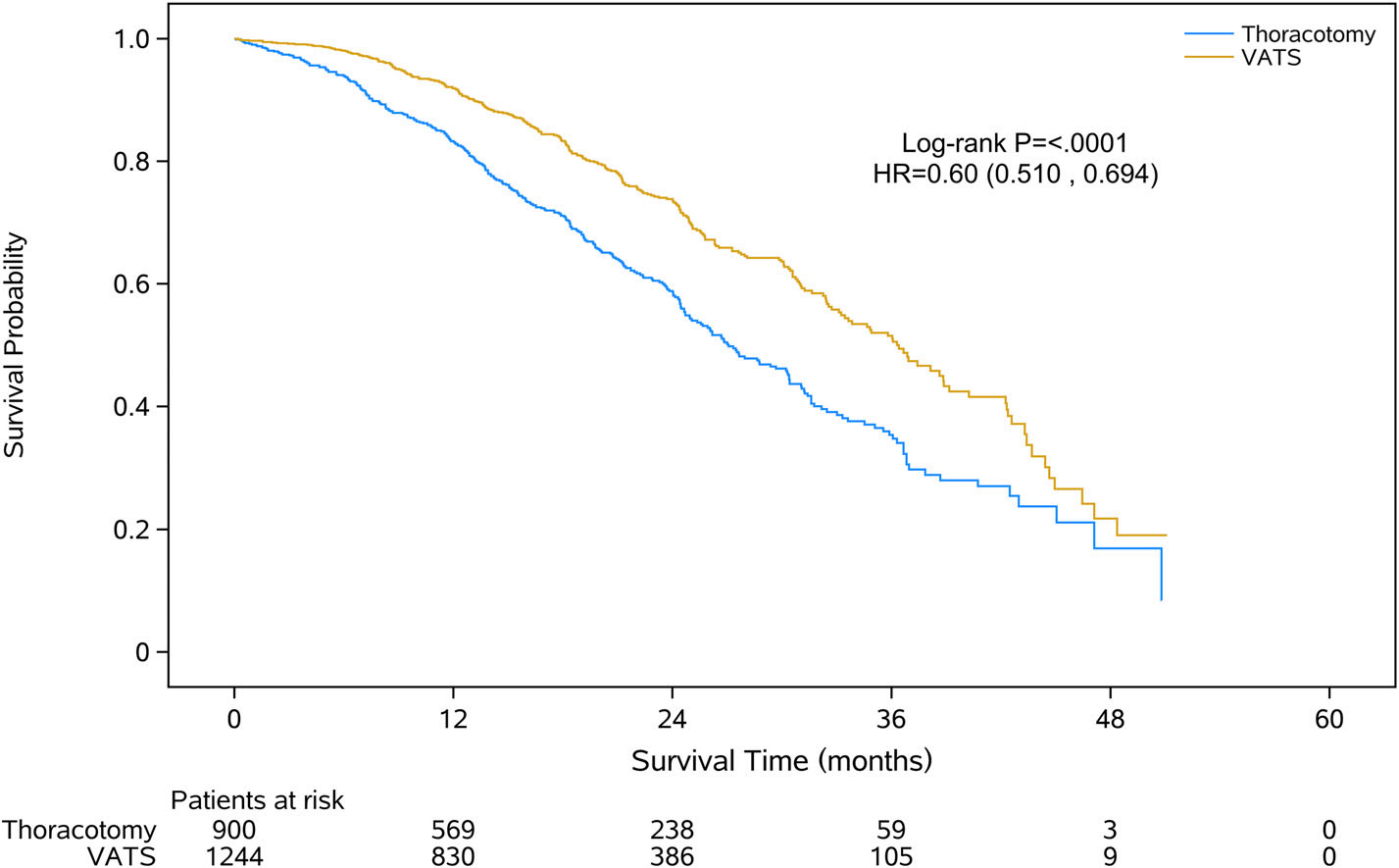
Kaplan‐Meier analysis of overall survival of resected pN2 NSCLC patients undergoing VATS or open lobectomy. VATS, video‐assisted thoracoscopic surgery.

Among 2144 patients, 305 (24.5%) patients managed by VATS lobectomy died during a median follow‐up of 16.7 months, while 344 (38.2%) patients died in the open lobectomy group during a median time interval of 15.6 months. Survival analysis based on log‐rank test indicated that VATS lobectomy was associated with significantly better overall survival of patients with surgically resected pN2 NSCLC (*P* < 0.0001, Fig 2).

On univariable analysis, almost all patient and tumor characteristics were associated with overall survival of the patients (*P* < 0.05, Table 3). After adjustment in a multivariate COX model, surgical approach of thoracotomy, age > 70 years, smoking, histology other than squamous cell carcinoma or adenocarcinoma, advanced pT stage of T2–T4, multiple N2 station involvement and perioperative transfusion were major factors independently associated with worse overall survival (Table 3).

**Table 3:** COX proportion hazard regression analysis of overall survival of the resected pN2 NSCLC patients

	Univariate analysis			Multivariate analysis		
Variable	HR	95% CI	*P*‐value	HR	95% CI	*P*‐value
VATS vs. open lobectomy	0.60	0.510, 0.694	<0.0001	0.75	0.621, 0.896	0.0017
Age ≥ 70 (vs.<70 years)	1.21	0.933, 1.559	0.0115	1.39	1.046, 21.858	0.0233
Female sex (vs. male)	0.67	0.564, 0.789	<0.0001	0.91	0.700, 1.317	0.7152
Smoking history (yes vs. no)	1.56	1.328, 1.822	<0.0001	1.45	1.137, 1.852	0.0028
Pathology			<0.0001			0.0002
Adenocarcinoma	Ref			Ref		
Squamous cell carcinoma	1.44	1.197, 1.722	<0.0001	1.21	0.978, 1.503	0.0789
Others	1.51	1.155, 1.974	0.0026	1.81	1.359, 2.407	<0.0001
Tumor location (right vs. left)	1.10	0.940, 1.292	0.2312	1.09	0.911, 1.301	0.3497
pT stage			<0.0001			0.0002
T1	Ref			Ref		
T2	1.49	1.171, 1.905	0.0012	1.41	1.097, 1.812	0.0073
T3	2.31	1.721, 3.113	<0.0001	1.86	1.361, 2.539	<0.0001
T4	2.54	1.769, 3.647	<0.0001	2.03	1.392, 2.964	0.0002
Single N2 station (vs.multiple station)	0.75	0.642, 0.876	0.0003	0.67	0.566, 0.803	<0.0001
Perioperative transfusion	1.51	1.193, 1.920	0.0006	1.40	1.079, 1.817	0.0114
Perioperative complications	0.93	0.678, 1.280	0.6612	1.01	0.725, 1.403	0.9598
Surgical margin (R1/R2 vs. R0)	1.62	1.202, 2.192	0.0016	1.42	0.990, 2.028	0.0570

pT, pathological T stage; VATS, video‐assisted thoracoscopic surgery.

**Figure 3:**
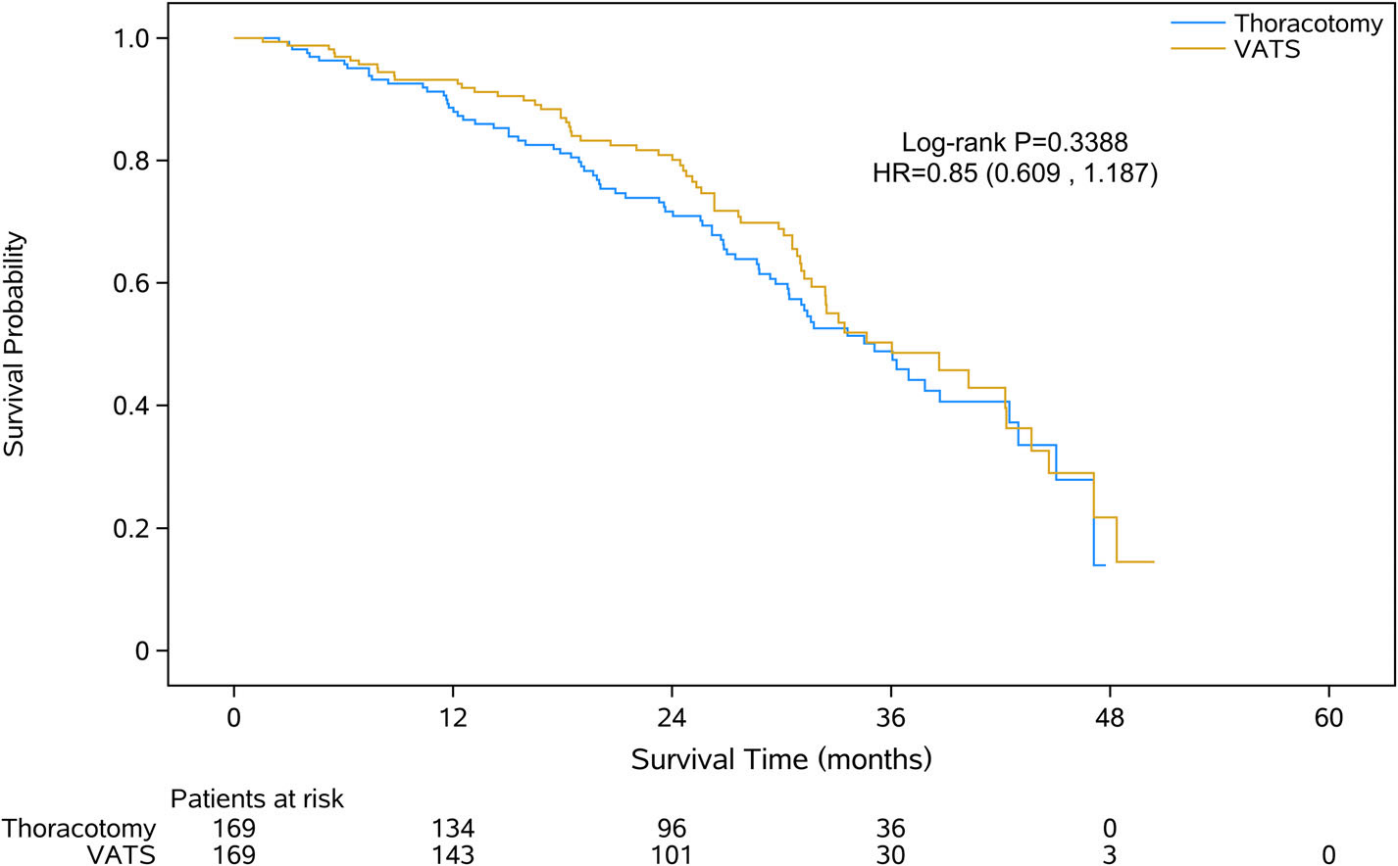
Kaplan‐Meier analysis of overall survival of resected pN2 NSCLC patients undergoing VATS or open lobectomy after propensity‐score matching. VATS, video‐assisted thoracoscopic surgery.

After propensity‐score matching (PSM) analysis, 169 patients were extracted from either group, respectively (Table 4). Blood loss in patients was less in the VATS group than the thoracotomy group, but with longer surgical time (Table 5). No difference in survival was found in the VATS group compared with the thoracotomy group (Fig 3).

**Table 4:** Baseline characteristics of resected pN2 NSCLC patients after propensity‐score matching

Variables	Thoracotomy (*n* = 169)	VATS (*n* = 169)	*P*‐value
Age (mean ± SD)	57.79 (± 8.574)	58.78 (± 9.252)	0.3031
Sex (*n*, %)			0.9069
Female	53 (31.4%)	54 (32%)	
Male			
Pathology			0.4257
Adenocarcinoma	107 (63.3%)	110 (65.1%)	
Squamous cell carcinomas	42 (24.9%)	46 (27.2%)	
Others	20 (11.8%)	13 (7.7%)	
Grade			0.4175
Poor	34 (27.2%)	26 (20.2%)	
Moderate	84 (67.2%)	95 (73.6%)	
Well	7 (5.6%)	8 (6.2%)	

VATS, video‐assisted thoracoscopic surgery.

**Table 5:** Perioperative outcomes of resected pN2 NSCLC patients after propensity‐score matching

Variables	Thoracotomy (*n* = 169)	VATS (*n* = 169)	*P*‐value
Operation time, minutes	178.4 (± 59.76)	190.9 (± 63.01)	0.0404
Estimated blood loss, mL	363.4 (± 566.28)	222.0 (± 326.49)	<0.0001
Postoperative drainage, days	7.1 ± 4.9	6.5 ± 4.5	0.2627
Surgical margin, *n* (%)^†^			0.2699
R0	159 (94.6%)	164 (97.0%)	
R1/R2	9 (5.4%)	5 (3.0%)	
Perioperative complications, *n* (%)	1 (0.6%)	5 (3.0%)	0.215
Perioperative mortality, *n* (%)			
30‐day	0	0	
90‐day	2 (1.2%)	3 (1.8%)	0.652

VATS, video‐assisted thoracoscopic surgery.

## Discussion

Using the largest cohort of surgically resected pN2 NSCLC patients reported to date, the present study showed the current status of patients undergoing VATS lobectomy when compared with conventional thoracotomy in China. These observations were consistent with previous studies, which indicated comparable outcomes with the VATS approach for the treatment of pN2 NSCLC.^14^, ^16^ Meanwhile, the shorter duration of chest tube drainage and postoperative hospital stay were also reported without compromising the overall survival of patients.^13^

VATS lobectomy, when compared with the open procedure, has long been established to be associated with better perioperative outcome (shorter hospital stay, fewer complications, etc) in early stage NSCLC patients.^8^ In addition, it has also been suggested to be a powerful predictor for clinical outcome.^23^ A recent phase III multicenter randomized controlled trial of VATS lobectomy versus thoractomy in stage I–III NSCLC patients showed equivalent oncological efficacy between the two surgical approaches.^24^ However, despite increasing evidence that shows acceptable outcomes of minimally invasive VATS lobectomy for pN2 NSCLC, conversion to conventional thoracotomy is still considered when unconfirmed N2 is suspected. Several factors, including inadequate experience during training,^25^ poor compliance of general surgeons with the lung cancer guideline,^26^, ^27^ and inferior oncological effectiveness (eg, lymph node assessment),^10^, ^18^ may contribute to the major concerns of VATS for pN2 NSCLC. In the real‐world consecutive patient experience, all pN2 NSCLC patients were surgically managed with VATS or open lobectomy by board‐certified thoracic surgeons from the 10 tertiary hospitals in China. Patients were treated under more advanced care system and standard surgical techniques, the effects of treatment related mortality might be well controlled.

The representative of this large cohort of patients included in this study provide us with a better chance of exploring the perioperative and long term outcome of VATS lobectomy in surgically resectable pN2 NSCLC patients. The estimated three‐year overall survival rates were 68.7% in the VATS group and 48.0% in the thoracotomy group (*P* < 0.0001), which was well within the ranges reported in previous studies (41.3%–87.4% for VATS approach and 25.5%–81.6% for thoracotomy approach).^6^, ^13^, ^14^, ^28^

Considering the imbalance of baseline characteristics between VATS and thoracotomy patients, especially in thoracic T stage and the application of neoadjuvant treatment that would affect selection of surgical procedure, a PSM was performed to reduce the biases and further explore clinical outcomes of these two groups. VATS showed less blood loss and longer operation time. In addition, no survival advantage was found in either group. These results were consistent with previous studies,^12^, ^13^, ^14^ and suggest that VATS might be a considerable choice, which would not decrease the clinical outcomes, for pN2 NSCLC compared with thoracotomy.

The extent of lymph node assessment is generally considered as a major issue in clinical patient management and prognostic prediction of pN2 NSCLC.^29^ It also emerged as the major concern regarding the inferior oncological effectiveness of minimally invasive technology, primarily due to inadequate mediastinal lymph node dissection. Previous studies have indicated that the number of involved lymph node stations (single vs. multiple stations) was considered as the prognostic factor for overall patient survival.^30^ However, a meta‐analysis revealed that there was no difference in N2 stations harvested using VATS or an open approach.^18^ Further, VATS treated patients showed more favorable survival outcomes versus thoracotomy with comparative N2 involved stations.^31^ It follows that VATS lobectomy could achieve similar outcomes to thoracotomy for pN2 NSCLC, while more careful and focused lymph node sampling or dissection was achieved. In the current study, patients in the two groups had similar OS with no statistical difference in N2 station involved (*P* = 0.0643) after PSM analysis,. It confirms that VATS may be a considerable choice for pN2 NSCLC by suitable selection and surgical techniques.

The selection bias intrinsic to retrospective design is the primary limitation of this study. Although tremendous efforts have been made, potential differences may still exist that obscure the oncological efficacy of treatment strategies. In addition, the study was composed of multicenter data, and inherent biases were unavoidable. For instance, postoperative adjuvant therapies were not available for all our patients (70.3%) since only the data from surgical departments were obtained. Despite this, since the adjuvant therapies have been routinely recommended and delivered in surgically managed pN2 patients, we hypothesized here that the postoperative patient care was active and comparable between the two groups. Also, despite PSM was performed, there was still a trend of more single station involved patients in VATS treated group. However, the present study was the realworld experience from multiple centers, retrospective design restricts further analysis of the oncogenic effects under absolutely balanced conditions. In addition, only data from the tertiary hospitals with experienced surgeons and advanced medical facilities were available. Also, the heterogeneity in international practice patterns must be acknowledged. In China, upfront surgery is considered acceptable as first‐line therapy for selected clinical stage IIIA (N2) NSCLC.^20^ However, it apparently falls outside the standard of care in the USA, where induction therapy is primarily recommended before surgery.^32^ Therefore, the current findings cannot be easily generalized and should be interpreted with caution. Last but not least, in this study we only reported the all‐cause mortality but not the cancer specific mortality.

In conclusion, our study showed that minimally invasive VATS lobectomy resulted in perioperative outcome and overall survival comparable to conventional thoracotomy. VATS lobectomy might be an effective alternative to the conventional open procedure for patients with surgically resectable pN2 NSCLC. However, randomized controlled trials are needed for further clarification in the future.

## Disclosure

The authors declare that there are no conflicts of interest.
